# Diverse, Abundant, and Novel Viruses Infecting the Marine *Roseobacter* RCA Lineage

**DOI:** 10.1128/mSystems.00494-19

**Published:** 2019-12-17

**Authors:** Zefeng Zhang, Feng Chen, Xiao Chu, Hao Zhang, Haiwei Luo, Fang Qin, Zhiqiang Zhai, Mingyu Yang, Jing Sun, Yanlin Zhao

**Affiliations:** a Fujian Provincial Key Laboratory of Agroecological Processing and Safety Monitoring, College of Life Sciences, Fujian Agriculture and Forestry University, Fuzhou, Fujian, China; b Institute of Marine and Environmental Technology, University of Maryland Center for Environmental Science, Baltimore, Maryland, USA; c Simon F. S. Li Marine Science Laboratory, School of Life Sciences and State Key Laboratory of Agrobiotechnology, The Chinese University of Hong Kong, Shatin, Hong Kong SAR, China; d Yellow Sea Fisheries Research Institute, Chinese Academy of Fishery Sciences, Qingdao, Shandong, China; University of Hawaii at Manoa

**Keywords:** *Roseobacter*, RCA lineage, RCA phages, viromics

## Abstract

Many major marine bacterial lineages such as SAR11, *Prochlorococcus*, SAR116, and several *Roseobacter* lineages have members that are abundant, relatively slow-growing, and genome streamlined. The isolation of phages that infect SAR11 and SAR116 have demonstrated the dominance of these phages in the marine virosphere. However, no phages have been isolated from bacteria in the *Roseobacter* RCA lineage, another abundant group of marine bacteria. In this study, seven RCA phages that infect three different RCA strains were isolated and characterized. All seven RCA phages belong to the *Podoviridae* family and have genome sizes ranging from 39.6 to 58.1 kb. Interestingly, three RCA phages (CRP-1, CRP-2, and CRP-3) show similar genomic content and architecture as SAR116 phage HMO-2011, which represents one of the most abundant known viral groups in the ocean. The high degree of homology among CRP-1, CRP-2, CRP-3, and HMO-2011 resulted in the contribution of RCA phages to the dominance of the HMO-2011-type group. CRP-4 and CRP-5 are similar to the Cobavirus group roseophages in terms of gene content and organization. The remaining two RCA phages, CRP-6 and CRP-7, show limited genomic similarity with known phages and represent two new phage groups. Metagenomic fragment recruitment analyses reveal that these RCA phage groups are much more abundant in the ocean than most existing marine roseophage groups. The characterization of these RCA phages has greatly expanded our understanding of the genomic diversity and evolution of marine roseophages and suggests the critical need for isolating phages from the abundant but “unculturable” bacteria.

**IMPORTANCE** The RCA lineage of the marine *Roseobacter* group represents one of the slow-growing but dominant components of marine microbial communities. Although dozens of roseophages have been characterized, no phages infecting RCA strains have been reported. In this study, we reported on the first RCA phage genomes and investigated their distribution pattern and relative abundance in comparison with other important marine phage groups. Two of the four RCA phage groups were found closely related to previously reported SAR116 phage HMO-2011 and Cobavirus group roseophages, respectively. The remaining two groups are novel in the genome contents. Our study also revealed that RCA phages are widely distributed and exhibit high abundance in marine viromic data sets. Altogether, our findings have greatly broadened our understanding of RCA phages and emphasize the ecological and evolutionary importance of RCA phages in the marine virosphere.

## Abstract (precis)

The RCA lineage of the marine *Roseobacter* group represents one of the slow-growing but dominant components of marine microbial communities. Although dozens of roseophages have been characterized, no phages infecting RCA strains have been reported. In this study, we reported on the first RCA phage genomes and investigated their distribution pattern and relative abundance in comparison with other important marine phage groups. Two of the four RCA phage groups were found closely related to previously reported SAR116 phage HMO-2011 and Cobavirus group roseophages, respectively. The remaining two groups are novel in the genome contents. Our study also revealed that RCA phages are widely distributed and exhibit high abundance in marine viromic data sets. Altogether, our findings have greatly broadened our understanding of RCA phages and emphasize the ecological and evolutionary importance of RCA phages in the marine virosphere.

## INTRODUCTION

Viruses are abundant and infectious to microorganisms in the sea, outnumbering bacteria by an order of magnitude (1, 2). The majority of marine viruses are bacteriophages whose hosts (bacteria) are the most abundant living organisms in nature (1, 3). Bacteriophages impact the structure and function of the microbial community and thus have a major influence on the ocean biogeochemical cycles through diverse phage-host interactions (1–3). Although many marine phages have been isolated, most hosts used for phage isolation are readily cultivated and fast-growing in rich culture medium. For decades, microbial ecologists have been challenged by the fact that a large majority of bacteria in seawater are difficult to cultivate in the laboratory (4). Therefore, our understanding of marine virology is greatly hindered by the lack of isolated phages from broader bacterial groups in the ocean. From the perspective of viromic studies, a major challenge has always been the low percentage of identifiable viral sequences in the viromic databases, or viral “dark matter” (5–7). The large portion of unknown sequences in marine viromes is believed to be due to the lack of known viruses isolated from diverse microbial groups (6, 8). Despite the gap between viral isolation and viromics, the isolation of some important marine phages, such as SAR116 phage and SAR11 phages (pelagiphages), has greatly facilitated the interpretation of marine viromic data sets (9, 10). SAR11 and SAR116 are the two most abundant and widespread bacterioplankton groups in the ocean (11–13), but the isolates of SAR11 and SAR116 display low growth rates and streamlined genomes compared to other cultured representatives from diverse marine bacterioplankton groups (10, 14–16). The isolation of pelagiphages and SAR116 phage demonstrated that phages infecting abundant but relatively slow-growing marine bacteria make up a significant portion of marine viruses in the ocean (9, 10). In addition, phage isolation provides not only genomic information but also morphological and infectious data.

Members of the *Roseobacter* group in *Alphaproteobacteria* are abundant, widespread, and diverse and play important biogeochemical roles in the marine environment (17, 18). Although many roseobacters have been isolated using solid culture media and are readily cultivated in the laboratory, natural *Roseobacter* populations differ systematically from their cultured representatives (19). Four dominant *Roseobacter* lineages including RCA (also called NAC11-3 or DC5-80-3), CHAB-I-5, SAG-O19, and NAC11-7 remain largely uncultivated and poorly studied (20). These four *Roseobacter* lineages together account for up to >60% of the global pelagic roseobacters (20). Among these four *Roseobacter* lineages, RCA is the largest cluster of the *Roseobacter* group that can exceed the SAR11 clade in some oceanic regions (21–23). RCA is among the most abundant marine bacterioplankton groups, comprising up to 30% of the total bacterioplankton in the temperate and polar ocean regions and up to 35% of total bacterioplankton with the highest abundance in the Southern Ocean (20–23). In general, RCA members are difficult to cultivate using traditional methods. At present, five RCA members have been isolated using the dilution method (21, 24, 25), and only Planktomarina temperata RCA23 has been fully sequenced and physiologically characterized (21, 25, 26). The genome of RCA23 is streamlined, an indication of oligotrophic adaptation (26).

Currently, more than 30 phages that infect representatives of several major *Roseobacter* clusters have been reported (27). All of these roseophages were isolated from the *Roseobacter* strains that can grow in rich culture media and have high growth rates, such as *Roseobacter* SIO67, Ruegeria pomeroyi DSS-3, Roseobacter denitrificans OCh114, *Sulfitobacter* spp., and Dinoroseobacter shibae DFL 12 (27–32). Metagenomic analyses suggested that homologs of some roseophages were widespread in the ocean, while most of the isolated roseophages were not reported to make up a significant portion of viromic sequences in the ocean. The low abundance of these roseophages is likely associated with low concentrations of host cells in the natural environment. The *Roseobacter* group contains diverse members, some of which are adaptable to environmental changes and can be readily cultured, while others are abundant and often restricted to low-nutrient environments, such as the RCA lineage. Little is known regarding phages that infect the abundant but slow-growing roseobacters (27). There are multiple challenges associated with isolating phages that infect RCA bacteria. RCA bacteria grow only in diluted media and do not reach high cell densities (21, 24, 25). Furthermore, they do not grow well in solid media, eliminating the possibility of using plaque assay for phage isolation.

In this study, three different RCA bacteria closely related to Planktomarina temperata RCA23 were isolated from the coastal water of Pingtan Island, China. We intended to isolate phages that infect these newly isolated RCA strains. A total of seven RCA phages were isolated and purified. These phages were characterized in terms of their morphology, cross-infectivity, genome sequences, and viromic fragment recruitment. Our results show that RCA phages are abundant and diverse in the ocean and contain many genome types that were not previously recognized.

## RESULTS AND DISCUSSION

### Host strains.

Three *Roseobacter* strains, FZCC0023, FZCC0040, and FZCC0042, were isolated in 2017 from the coastal water of Pingtan Island, Fujian, China, using the dilution-to-extinction method (16). Phylogenetic analysis based on the 16S rRNA gene sequences suggests that FZCC0023, FZCC0040, and FZCC0042 all belong to the RCA cluster (see Fig. S1a in the supplemental material). In terms of the 16S rRNA gene (rDNA) sequence, FZCC0040 and FZCC0042 are 100% identical to RCA23, while FZCC0023 has 2 nucleotide mismatches with RCA23. However, these RCA strains can be distinguished from RCA23 and each other based on their 16S-23S rDNA intergenic spacer (ITS) sequences, suggesting that they are closely related but distinct RCA members (see Fig. S1b in the supplemental material). All three RCA strains grow slowly and reach low cell densities in natural seawater-based medium (see Fig. S1c in the supplemental material). They can neither grow in artificial seawater-based medium nor form visible colonies on solidified media. Further details are provided in Text S1 in the supplemental material.

10.1128/mSystems.00494-19.1 TEXT S1 Supplemental methods and results. Download Text S1, DOCX file, 0.02 MB. Copyright © 2019 Zhang et al. 2019 Zhang et al. This content is distributed under the terms of the Creative Commons Attribution 4.0 International license.

10.1128/mSystems.00494-19.2 FIG S1 Phylogenetic analysis and growth characteristics of three RCA isolates. (a) Phylogenetic relationship between three RCA isolates and the representatives belonging to the *Roseobacter* group of *Alphaproteobacteria* based on 16S rRNA gene. (b) Phylogenetic analysis of our RCA isolates and four RCA representatives based on the ITS sequences. (c) Growth curves of three RCA strains in natural seawater-based medium. Data points represent the mean values from biological triplicates, and error bars represent the standard deviation. Download FIG S1, TIF file, 2.5 MB. Copyright © 2019 Zhang et al. 2019 Zhang et al. This content is distributed under the terms of the Creative Commons Attribution 4.0 International license.

### Isolation and general features of RCA phages.

**FIG 1:**
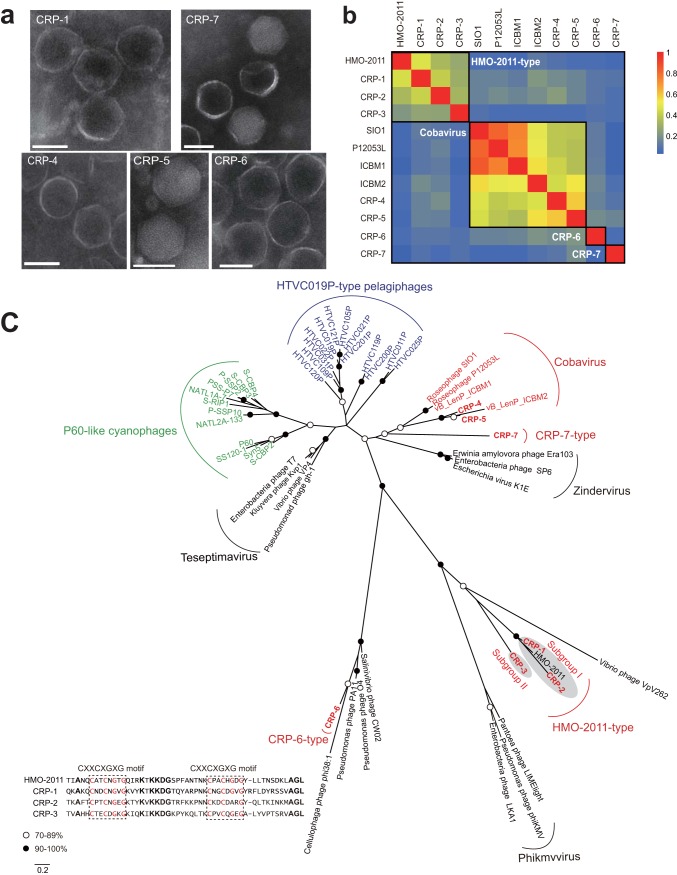
(a) Transmission electron microscopy images of selected representative RCA phages from each group (bars, 50 nm). (b) Heatmap presentation of shared genes of newly isolated RCA phages and five related marine phages (HMO-2011, SIO1, P12053L, ICBM1, and ICBM2). The genome similarity between the phages refers to the heatmap bar on the right. Phages in the same group are boxed. (c) Unrooted maximum-likelihood phylogenetic tree of phage DNA polymerases constructed with conserved polymerase domains. Black and white circles indicate nodes with 90 to 100% and 70 to 89% bootstrap support, respectively. Roseophages, cyanophages, and pelagiphages are shown in red, green, and blue, respectively. Scale bar represents amino acid substitutions per site. The alignment of the partial sequence of the DnaJ central domain from HMO-2011 and three RCA phages is presented. Two CXXCXGXG motifs are boxed. Conserved residues in the two CXXCXGXG motifs are in red. Other conserved residues are in bold.

**FIG 2:**
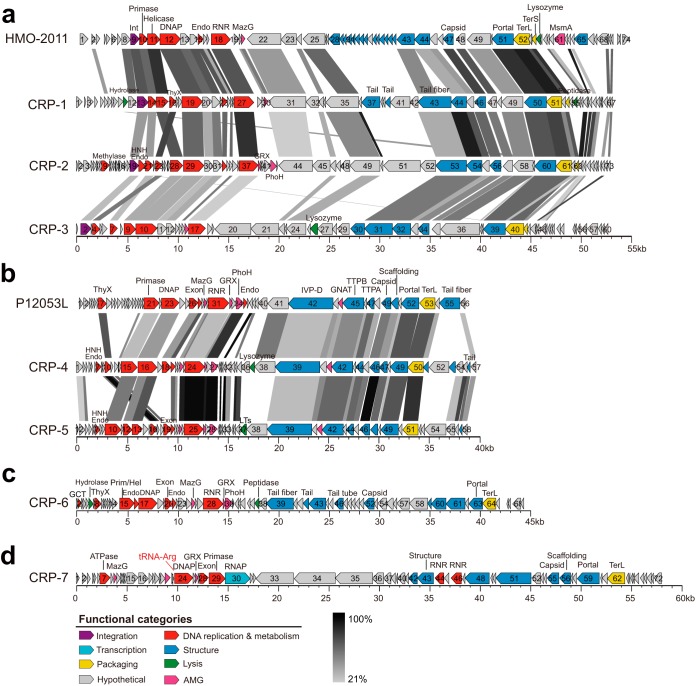
Genomic organization and comparison of RCA phages. (a) HMO-2011-type phages. (b) Cobavirus group roseophages. (c) CRP-6. (d) CRP-7. ORFs are depicted by leftward or rightward arrows according to their transcription direction. ORFs are color coded according to putative biological function. Scale color bar indicates amino acid identities between genes. tRNAs are shown in red. Abbreviations: Int, integrase; RNAP, RNA polymerase; SSB, single-stranded DNA binding protein; Endo, endonuclease; DNAP, DNA polymerase; Exon, exonuclease; TerS, terminase, small subunit; TerL, terminase, large subunit; ThyX, thymidylate synthase; MazG, MazG nucleotide pyrophosphohydrolase domain protein; RNR, adenosylcobalamin-dependent ribonucleoside-triphosphate reductase; GNAT, GCN5-related *N*-acetyltransferase, acetyltransferase family; IVP-D, internal virion proteins D; TTPA, tail tubular protein A; TTPB, tail tubular protein B; GRX, glutaredoxin; GCT, glycerol-3-phosphate cytidylyltransferase; LTs, lytic transglycosylase.

**FIG 3:**
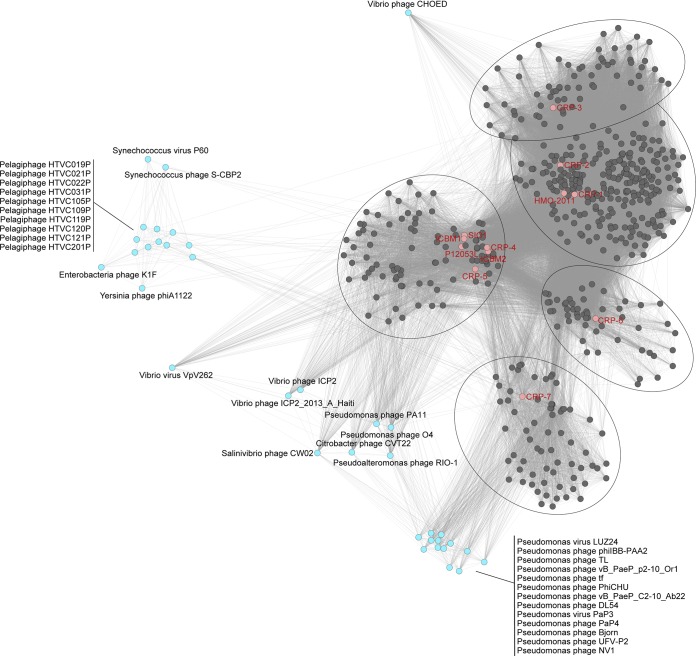
Gene-content-based viral network of RCA phages and related bacteriophages and environmental viral contigs. Nodes represent phages, and the weight of each edge represents the distance between two phages based on the similarity score, with a cutoff of ≥1. For clarity, viral genomic contigs that were not grouped with the RCA phages were excluded and bacteriophage genomes that do not link to the four viral clusters were excluded. Different viral clusters defined by vConTACT 2.0 are circled. Viral clusters generated by vConTACT2 are provided in Table S2 in the supplemental material.

Seven phages (CRP-1, CRP-2, CRP-3, CRP-4, CRP-5, CRP-6, and CRP-7) that infect the three abovementioned RCA strains (here referred to as RCA phages) were isolated from different locations ranging from temperate (Bohai Sea, China, and Osaka Bay, Japan) to subtropical (Pingtan coast, China) regions (Table 1). All seven RCA phages have short tails and icosahedral capsids (Fig. 1a); thus, they all belong to the *Podoviridae* family in the order *Caudovirales.* Except for CRP-7, the RCA phages did not cross-infect other RCA strains beyond the original host (Table 1). CRP-7, which was originally isolated from FZCC0042, was also able to infect FZCC0040. Considering the high 16S rRNA gene sequence identity of the tested RCA strains, this result suggests that these RCA phages have a very narrow host range. The complete genomes of these seven RCA phages were sequenced and assembled, and their genome sizes vary from 39.6 to 58.1 kb. The G+C content of these phages ranges from 40.3% to 49.7%, lower than that of their hosts (50% to 53%). The RCA phages appear to have a relatively lower G+C content than other sequenced roseophages (43% to 64%) (27). Except for CRP-7, the RCA phages do not contain tRNA sequences. Comparative genomics analysis and gene-content-based network analysis categorized these 7 RCA phages into four distinct phage groups within the *Podoviridae* family (Fig. 1b and Fig. 2 and 3). Taken together, these results suggest that RCA roseobacters are subjected to phage infection by diverse podoviruses (non-N4-like podoviruses), a scenario different from the known roseophages that infect readily cultivated marine roseobacters (27). Among the 32 known roseophages, siphoviruses and N4-like podoviruses dominate the current isolates (27).

**TABLE 1:** General features of seven RCA phages analyzed in this study

Phage	Original host	Infectivity for host:			Source water^*a*^	Latitude	Longitude	Collection date	Genome size (bp)	% G+C	No. of ORFs	Accession no.
		FZCC0023	FZCC0040	FZCC0042								
CRP-1	FZCC0023	+^*c*^			Osaka Bay, Japan	N34°27′	E135°21′	August 2017	54,045	42.43	67	MK613343
CRP-2	FZCC0023	+			Pingtan coast, Taiwan Strait	N25°26′	E119°47′	May 2017	54,148	42.95	73	MK613344
CRP-3	FZCC0040	ND^*b*^	ND	ND	Yantai coast, Bohai Sea	N37°28′	E121°28′	May 2017	52,963	49.67	60	MK613345
CRP-4	FZCC0023	+			Yantai coast, Bohai Sea	N37°28′	E121°28′	May 2017	40,768	45.20	57	MK613346
CRP-5	FZCC0040		+		Pingtan coast, Taiwan Strait	N25°26′	E119°47′	May 2017	39,600	45.63	58	MK613347
CRP-6	FZCC0042			+	Pingtan coast, Taiwan Strait	N25°26′	E119°47′	May 2017	44,927	47.05	69	MK613348
CRP-7	FZCC0042		+	+	Yantai coast, Bohai Sea	N37°28′	E121°28′	May 2017	58,106	40.29	72	MK613349

a All seawater samples were collected from surface water.

b ND, not done.

c +, able to infect.

10.1128/mSystems.00494-19.9 TABLE S2 List of viral genomic contigs in each viral cluster in the network analysis. Download Table S2, XLSX file, 0.04 MB. Copyright © 2019 Zhang et al. 2019 Zhang et al. This content is distributed under the terms of the Creative Commons Attribution 4.0 International license.

### Close kinship between RCA phages and SAR116 phage HMO-2011.

Three RCA phages (CRP-1, CRP-2, and CRP-3) share a similar genomic content and arrangement with SAR116 phage HMO-2011, belonging to the HMO-2011-type group (Fig. 2a). HMO-2011, which infects a SAR116 bacterium, “*Candidatus* Puniceispirillum marinum” strain IMCC1322, represents one of the most abundant known phage groups in marine viromes (10). CRP-1, CRP-2, and CRP-3 have 30, 25, and 16 open reading frames (ORFs) homologous to genes in the HMO-2011 genome, respectively. About half of the shared genes in CRP-1 and CRP-2 are >50% identical in amino acid sequence to their counterparts in HMO-2011, while most shared genes in CRP-3 are <50% identical in amino acid sequences to their HMO-2011 counterparts. CRP-3 is clearly more distantly related to CRP-1, CRP-2, and HMO-2011 based on the comparative genome analysis. Thus, the HMO-2011-type group phages can be separated into two subgroups, with CRP-1, CRP-2, and HMO-2011 belonging to subgroup I and CRP-3 belonging to subgroup II. The shared genes within the HMO-2011-type group are primarily involved in the essential functions for the phage life cycle, including DNA metabolism and replication, phage structure, and DNA packaging, and they are arranged in a conserved order (Fig. 2a). There is no significant genomic feature that distinguishes these three RCA phages from SAR116 phage. Only three genes were observed to be exclusive to these three RCA phages, including a thymidylate synthase gene, a tail fiber gene, and a gene encoding an unknown-function protein (ORF47 in CRP-3). Phage tail fibers are responsible for host specificity. The variation in tail fiber genes between the RCA phages and the SAR116 phage suggests an adaptation of phages to different host systems. Although HMO-2011-type phages are among the most abundant known viral groups in the ocean (10), HMO-2011 has no counterparts among currently known phages. The high genomic homology among CRP-1, CRP-2, CRP-3, and HMO-2011 suggests a close kinship between the RCA phages and the SAR116 phage.

The close relationship between the three RCA phages and HMO-2011 is also evident based on the DNA polymerase gene, a gene that is particularly conserved among marine podoviruses (33). The amino acid sequences of the DNA polymerases of these three RCA phages are 40% to 67% identical to that of HMO-2011. The DNA polymerase gene phylogeny shows that CRP-1, CRP-2, and HMO-2011 cluster into one subgroup, while CRP-3 forms its own branch adjacent to this subgroup (Fig. 1c). The HMO-2011 DNA polymerase possesses an unusual domain architecture, with a partial DnaJ domain located between the exonuclease domain and the DNA polymerase domain (10). The DNA polymerases of these three RCA phages also exhibit this unusual domain structure and contain two CXXCXGXG motifs in the partial DnaJ domain (box in Fig. 1c). CRP-1, CRP-2, and CRP-3 all encode a tyrosine integrase upstream of the DNA replication and metabolism module, which shares 35% to 63% amino acid identity with the HMO-2011 integrase. Integrase genes typically occur in the genomes of temperate phages and are responsible for site-specific integration. The presence of an integrase suggests that these phages possibly undergo a lysogenic life cycle. The high-throughput sequencing analysis and PCR amplification of the integration sites verified that CRP-3 can be integrated into a tRNA-Met (CAT) site in the FZCC0040 genome (see Fig. S2a in the supplemental material), suggesting that CRP-3 reproduction occurs via lytic and lysogenic cycles. The core sequence overlaps the 3′ end of the host tRNA-Met gene (see Fig. S2b in the supplemental material). However, the integration sites of CRP-1 and CRP-2 in FZCC0023 have not yet been identified; thus, it is still unknown whether CRP-1 and CRP-2 also have a lysogenic life cycle.

10.1128/mSystems.00494-19.3 FIG S2 Integration of CRP-3 in the FZCC0040 genome. (a) CRP-3 and host FZCC0040 genes are shown in pink and blue, respectively. The core sequences are indicated by the arrows. PCR primers are indicated by red triangles. (b) The DNA sequences of all integration sites. CRP-3 sequences, FZCC0040 sequences, and the identical core sequences are shown in black, blue, and red, respectively. The tRNA genes are boxed. DNAP, DNA polymerase. Download FIG S2, TIF file, 1.6 MB. Copyright © 2019 Zhang et al. 2019 Zhang et al. This content is distributed under the terms of the Creative Commons Attribution 4.0 International license.

These results suggest that the HMO-2011-type group contains members that infect diverse bacterial hosts. It is interesting that within the HMO-2011-type subgroup I (at approximately genus level), members infecting SAR116 and RCA roseobacters are strikingly similar. The high sequence homology between the RCA phages and SAR116 phages raises a concern on potential overlaps among these phages on viromic fragment recruitment (see “Viromic fragment recruitment analyses of RCA phages” below).

It is generally observed that phages infecting closely related hosts appeared to be more closely related, and a recent phage phylogeny analysis suggested that phage genera usually infect bacteria within the same family (34). In contrast, in our study, closely related HMO-2011-type phages were observed to infect SAR116 and RCA bacteria, which belong to two distinct orders. SAR116 and RCA both possess high population densities (12, 13, 21, 22, 35) and are therefore among the most common phage hosts in the ocean. In addition, SAR116 and RCA display similar distribution patterns in the global ocean and are both predominant in temperate and polar oceans (13, 22, 35). Considering the high population densities of SAR116 and RCA, our results imply that common ancestors of these phages were more likely to collide with these abundant bacteria by chance and evolved to gain the ability to attach and take control of host machinery. Thus, the genome sequences of these RCA phages provide important clues for understanding the evolution and taxonomy of this important phage group.

### Two RCA phages are closely related to Cobavirus group roseophages.

CRP-4 and CRP-5 share similar genome content and architecture with the roseophages SIO1, P12053L, and ICBM1 and ICBM2, which infect *Roseobacter* SIO67, *Celeribacter* sp. strain IMCC12053, and *Lentibacter* sp. strain SH36, respectively (28, 36, 37) (Fig. 2b). Although these roseophages are related to the phages of the *Autographivirinae* subfamily from an evolutionary perspective, they were previously classified as an unassigned *Podoviridae* group because they all lack a phage-encoded RNA polymerase (38). Recently, a new Cobavirus group was established after the isolation of three marine cobaviruses (cobalamin dependent) (37). CRP-4 and CRP-5 also lack an RNA polymerase gene and contain a cobalamin-dependent ribonucleotide reductase (RNR) gene (Fig. 2b), and they can be classified as new members of the Cobavirus group based on their genome synteny (Fig. 1b and Fig. 2b). Most of the shared genes within the Cobavirus group are located in the DNA replication module, including genes predicted to encode a thymidine synthase, a DNA primase, a T7-like DNA polymerase, an endonuclease, and a ribonucleotide reductase. Other homologous genes encode proteins involved in the phage virion structure and DNA packaging, such as the coat protein, portal protein, tail proteins, and the large subunit of terminase (Fig. 2b). The DNA polymerase gene phylogeny indicates that CRP-4 and CRP-5 are clustered with four other Cobavirus roseophages, as expected (Fig. 1c).

### CRP-6 and CRP-7 represent two novel phage groups.

CRP-6 shares limited homology with other known phage isolates. Nearly 30% of the ORFs in CRP-6 can be assigned putative biological functions (Fig. 2c). Although CRP-6 appears to be closely related to *Cellulophaga* phage phi38:1 and *Salinivibrio* phage CW02 based on the DNA polymerase phylogeny (Fig. 1c), CRP-6 shares only a few genes with phi38:1 and CW02 at the genomic level. Therefore, in this case, the DNA polymerase gene phylogeny does not reflect the genomic evolution of CRP-6. The genome of CRP-6 appears to be highly mosaic, as it shares the DNA replication genes with some podoviruses but shares its structural genes with other types of phages. For example, the CRP-6 primase/helicase gene is most closely related to those found in Cobavirus roseophages, the DNA polymerase gene of CRP-6 is most closely related to that of *Cellulophaga* phage phi38:1, the capsid gene of CRP-6 is similar to that of roseosiphovirus RDJL Phi 1, and the tail fiber gene of CRP-6 is similar to that of cyanomyovirus S-CAM7. CRP-6 encodes a terminase large-subunit-like protein with homologs found in some bacterial genomes.

CRP-7 has the largest genome size among the seven RCA phage isolates (Table 1). The CRP-7 genome is 58.1 kb in length, consisting of 73 predicted ORFs and a tRNA-Arg (TCT) gene. Similarly to CRP-6, CRP-7 also exhibits no significant genomic synteny with other known phage isolates. Of the 73 predicted ORFs, 35 have homologs in the NCBI-RefSeq database and only 18 have assigned functions (Fig. 2d). Functional predictions for the annotated ORFs were predominantly associated with phage DNA replication and virion morphogenesis. CRP-7 contains a suite of DNA replication genes with divergent similarity to the phages in the *Podoviridae* family. The DNA polymerase of CRP-7 encoded by ORF24 is mostly related to the DNA polymerase in Cobavirus roseophages. ORF30 was predicted to encode a DNA-dependent RNA polymerase, having a distant relationship to the RNA polymerase in members of the *Autographivirinae* subfamily (<25% amino acid identity). Additionally, several structural genes were predicted from the CRP-7 genome, most of which show weak homology to structural proteins in other *Podoviridae* phages. The gene encoding the terminase large subunit in CRP-7 is also very divergent, showing similarity with some bacterial terminase genes and a distant relationship to the terminases in some T4-like cyanomyoviruses. The DNA polymerase gene phylogeny reveals that CRP-7 is distantly related to other known marine podoviruses, forming a branch near Cobavirus roseophages (Fig. 1c).

### Novelty of the RCA phages.

We built a gene-content-based network to illustrate the relationship of RCA phages to other known bacteriophages and metagenomic viral fragments. Distinct viral clusters were generated by using vConTACT2, corresponding to approximately genus-level grouping (Fig. 3). In agreement with the genomic comparative analysis and the DNA polymerase gene phylogeny, CRP-1 and CRP-2 were grouped with HMO-2011, whereas CRP-3 formed another cluster nearby. CRP-4 and CRP-5 were clustered with Cobavirus roseophages. The remaining two viral clusters were represented by CRP-6 and CRP-7, respectively. Notably, a number of metagenomic viral contigs were placed into these viral clusters, suggesting that close relatives of these phage groups are widely distributed in the ocean (Fig. 3). Furthermore, these viral clusters show only weak relationships with some *Podoviridae* phages. The network analysis further supports the idea that phages infecting RCA can be diverse and new to our current collection of marine phages (Fig. 3).

Interestingly, all seven RCA phages belong to the *Podoviridae.* It is somewhat surprising that RCA podoviruses show greater genomic diversity than the cyanopodoviruses that infect marine *Synechococcus* and *Prochlorococcus* (39), considering that the three RCA host strains that we used are closely related. Similarly, high genomic diversity was also reported on marine *Cellulophaga* podoviruses isolated using closely related hosts (40).

### RCA phage AMGs and genes involved in other cellular processes.

All RCA phage genomes possess several auxiliary metabolic genes (AMGs), which are presumably of bacterial origin. AMGs play roles in the regulation of host metabolism during host infection and therefore benefit phage production (41). The AMGs identified from the seven RCA phage genomes are summarized in Table S1 in the supplemental material. All seven RCA phages harbor a gene encoding an adenosylcobalamin-dependent ribonucleoside triphosphate reductase (RNR) (PF02867.14), and all but one RCA phage harbor a gene encoding a thymidylate synthase, which is possibly involved in phage nucleotide metabolism. Another AMG involved in nucleotide metabolism is a putative DNA (cytosine‐5) methylase gene identified from CRP-2. DNA methylase is involved in cytosine residue methylation, commonly occurs in bacterial genomes, and has been also found in many phage genomes. This DNA methylase may possibly methylate the phage sequence and protect it against host restriction-modification (R-M) systems (42).

10.1128/mSystems.00494-19.8 TABLE S1 Summary of putative AMGs and content of genes involved in other cellular processes across seven roseophages. Download Table S1, DOCX file, 0.02 MB. Copyright © 2019 Zhang et al. 2019 Zhang et al. This content is distributed under the terms of the Creative Commons Attribution 4.0 International license.

Five RCA phages investigated in this study also possess a starvation-inducible protein gene (*phoH*), suggesting its importance in the phosphate metabolism of phage-infected roseobacterial cells. All but one RCA phage encodes a conserved MazG pyrophosphohydrolase domain (PF03819). The *mazG* gene was also identified in many marine phage genomes (10, 43–45). MazG proteins are involved in the regulation of bacterial survival under nutritional stress (46). MazG proteins can extend the period of cell survival, which is important for phage reproduction (47). It was hypothesized that phages encoding MazG proteins play an important role in phage propagation by maintaining host metabolism under stress (10, 43, 45).

Glutaredoxin genes were predicted from five RCA phage genomes. Glutaredoxins are redox proteins that have been shown to be involved in multiple cellular processes, such as oxidative stress response, deoxyribonucleotide synthesis, repair of oxidatively damaged proteins, protein folding, sulfur metabolism, and other redox-dependent signaling, by catalyzing glutathione-disulfide oxidoreductions (48). Glutaredoxins also serve as hydrogen donors in the redox reactions of the RNR catalysis (49). It was suggested that glutaredoxins may contribute to phage propagation by maintaining the cellular redox state of the host and by interacting with the phage-encoded ribonucleotide reductase (49).

Interestingly, CRP-4 and CRP-5 each encode a GCN5-related *N*-acetyltransferase (GNAT). GNATs catalyze the transfer of an acyl group from acyl coenzyme A (acyl-CoA) to an amino group of a wide range of substrates (50). GNATs are involved in diverse cellular processes, including carbohydrate and energy metabolism, nucleotide and amino acid metabolism, transcription, translation, cell differentiation, stress regulation, and many others (50). Because GNATs are integral to bacterial metabolism, it is thus suggested that CRP-4 and CRP-5 may regulate primary host metabolism to complete phage propagation through GNAT (50).

CRP-6 contains a gene encoding glycerol-3-phosphate cytidylyltransferase (GCT) (PF01467.25, CL0039), which is involved in the biosynthesis of teichoic acid and is required for bacterial cell wall biogenesis (51). The function of the GCT-encoding gene in CRP-6 remains unclear.

### Viromic fragment recruitment analyses of RCA phages.

**FIG 4:**
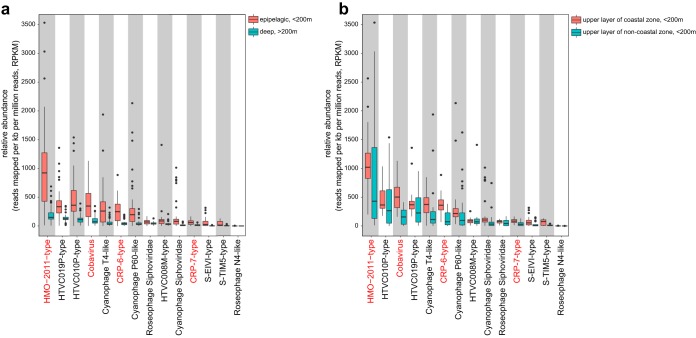
Box plots indicate the relative abundance of major phage groups in marine viromes. Note that the cyanosiphovirus group and roseosiphovirus group include all known cyanosiphovirus and roseosiphovirus types, respectively. The relative abundance (*y* axis) of each phage group (sorted according to its median relative abundance, *x* axis) in each virome data set was calculated and normalized as RPKM (number of reads recruited per kilobase pairs in average genome size per million of reads in the virome data set). The marine virome data sets were from the Pacific Ocean Virome (POV), Moore Virome Project (MVP), Indian Ocean Virome (IOV), Scripps Pier Virome (SPV), and Malaspina Expedition virome (ME). (a) Relative abundance of major phage groups in upper-ocean samples (<200 m) compared with their abundance in deep-ocean samples (>200 m). (b) Relative abundance of major phage groups in coastal water samples compared with their abundance in noncoastal water samples. The phage groups containing RCA phages are shown in red.

Metagenomic fragment recruitment analyses were performed to assess the distribution and relative abundance of represented RCA phage groups compared with other important marine phage groups. A total of 216 marine viromic data sets from the Pacific Ocean Virome (POV), Moore Virome Project (MVP), Scripps Pier Virome (SPV), Indian Ocean Virome (IOV), Malaspina Expedition virome (ME) and Global Ocean Viromes (GOV) were used for the recruitment analyses (see Data Set S1 in the supplemental material), which cover a wide range of marine habitats. The HMO-2011-type phage group was the most abundant known phage group in most of the marine viromes (Fig. 4; also see Fig. S3 in the supplemental material). These data are consistent with the previous finding that the phage type represented by HMO-2011 is among the most abundant viral groups in marine viromes (10). We also noticed that the reads assigned to subgroup II account for only approximately 10% of the total reads assigned to the HMO-2011-type group (data not shown), suggesting that HMO-2011-type subgroup II is not a dominant subgroup. Our results explain the high abundance of the HMO-2011-type group in the ocean, as known hosts of this group comprise SAR116 and RCA roseobacters. Due to the high sequence homology between the RCA phages and HMO-2011, it is obvious that the RCA phages, and probably other undiscovered marine phages, also contribute to the abundance and diversity of the HMO-2011-type group.

10.1128/mSystems.00494-19.4 FIG S3 Relative abundance of major phage groups in Global Ocean Viromes (GOV). Relative abundance (*y* axis) of each phage group (sorted according to its median relative abundance, *x* axis) in each virome data set was calculated and normalized as RPKM (number of reads recruited per kilobase pairs in average genome size per million of reads in the virome data set). The phage groups containing RCA phages are shown in red. Download FIG S3, TIF file, 3.9 MB. Copyright © 2019 Zhang et al. 2019 Zhang et al. This content is distributed under the terms of the Creative Commons Attribution 4.0 International license.

10.1128/mSystems.00494-19.10 DATA SET S1 Summary of metagenomic analyses, including viromic data sets and phage genomes used and the fragment recruitment results. Download Data Set S1, XLSX file, 0.1 MB. Copyright © 2019 Zhang et al. 2019 Zhang et al. This content is distributed under the terms of the Creative Commons Attribution 4.0 International license.

In the HMO-2011-type group, RCA phages CRP-1 and CRP-2 cannot be well separated from HMO-2011 based on the genome content and sequence identity (Fig. 1b and c; see also Fig. S4 in the supplemental material). In most shared ORFs, CRP-1 is more similar to HMO-2011 than to CRP-2 and CRP-3 (see Fig. S4 in the supplemental material). The read recruitment analysis shows that of the 97,684 reads assigned to the HMO-2011-type group in POV data sets, 78,349, 74,699, and 66,560 reads could be mapped to the genomes of HMO-2011, CRP-1, and CRP-2, respectively (see Fig. S5 in the supplemental material). Further, the recruited reads mapped to the genomes of HMO-2011, CRP-1, and CRP-2 show highly similar distribution patterns of sequence identities and bitscores (see Fig. S5 and S6 in the supplemental material). In addition, similarly to a previous study (10), the majority of recruitments were phage genes associated with DNA metabolism and replication, structure, and DNA packaging, which are conserved among CRP-1, CRP-2, and HMO-2011 (see Fig. S5 in the supplemental material). Taken together, the results of our study suggest that it is difficult to separate the viromic reads assigned to the SAR116 phages and RCA phages within the HMO-2011-type group.

10.1128/mSystems.00494-19.5 FIG S4 Percent amino acid identity of phages HMO-2011, CRP-2, and CRP-3 to the CRP-1 ORFs. Download FIG S4, TIF file, 0.1 MB. Copyright © 2019 Zhang et al. 2019 Zhang et al. This content is distributed under the terms of the Creative Commons Attribution 4.0 International license.

10.1128/mSystems.00494-19.6 FIG S5 Fragment recruitment plot of percent amino acid identity and bitscores of Pacific Ocean Virome (POV) reads against the ORFs of phages HMO-2011, CRP-1, and CRP-2. The total number of POV reads assigned to the HMO-2011-type group is 97,684. Numbers of reads mapped to HMO-2011, CRP-1, and CRP-2 are 78,349, 74,699, and 66,560, respectively. (a) HMO-2011. (b) CRP-1. (c) CRP-2. Download FIG S5, TIF file, 2.4 MB. Copyright © 2019 Zhang et al. 2019 Zhang et al. This content is distributed under the terms of the Creative Commons Attribution 4.0 International license.

10.1128/mSystems.00494-19.7 FIG S6 Box plot distributions of percent amino acid identity and bitscores of the POV reads assigned to HMO-2011-type group against each HMO-2011-type genome. Download FIG S6, TIF file, 0.8 MB. Copyright © 2019 Zhang et al. 2019 Zhang et al. This content is distributed under the terms of the Creative Commons Attribution 4.0 International license.

**FIG 5:**
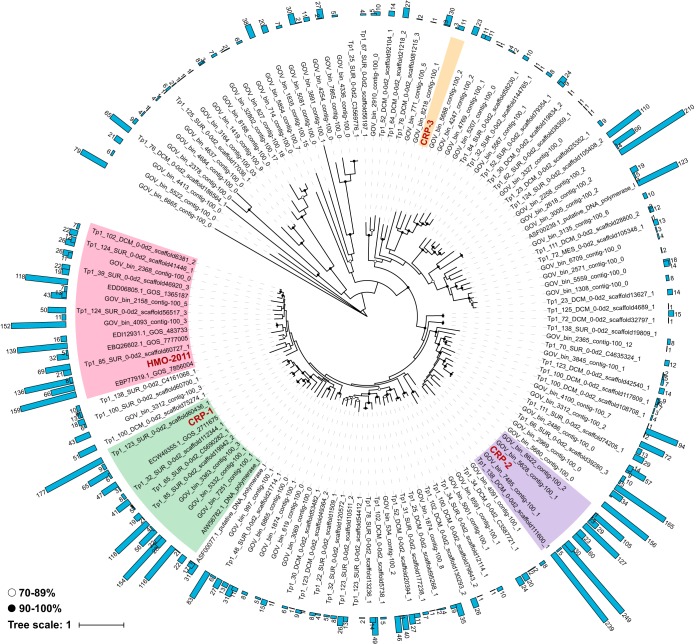
Phylogenetic placement of POV reads with homology to the DNA polymerase of HMO-2011-type phage. A total of 5,794 metagenomic reads were placed within the reference tree containing diverse homologs of HMO-2011-type DNA polymerase. DNA polymerase sequences closely related to the DNA polymerases of HMO-2011, CRP-1, CRP-2, and CRP-3 are indicated in pink, green, purple, and yellow, respectively (≥70% amino acid identity within group). The numbers of mapped viromic reads are shown by blue bars.

We also performed the phylogenetic placement of the translated DNA polymerase sequences from POV data sets. In total, 5,794 POV reads assigned to HMO-2011-type DNA polymerase were placed in the DNA polymerase reference tree (Fig. 5). The DNA polymerase gene phylogeny reveals that the HMO-2011-type group contains remarkably diverse subgroups (Fig. 5), and a significant fraction of these DNA polymerase reads were placed near the reference sequence for HMO-2011, CRP-1, and CRP-2 (Fig. 5), suggesting that SAR116 and RCA roseobacters are probably the two most important hosts for HMO-2011-type phages.

CRP-4 and CRP-5 are affiliated with the Cobavirus group, which is widely distributed and is among the top most abundant phage groups in marine viromes. Roseophage SIO1-related sequences were previously found to be abundant in the POV data sets (9), and Cobaviruses are ubiquitous in marine metagenomic data sets (37). In our analyses, the results show that the relative abundance of the Cobavirus group was comparable with those of the HTVC019P-type group and cyanophage T4-like group in the upper ocean viromic data sets of POV, MVP, SPV, and IOV (Fig. 4a), and the Cobavirus group was the second most abundant phage group in the coastal viromic data sets (Fig. 4b). In GOV data sets, we observed that the Cobavirus group exhibited significantly lower abundance (see Fig. S3 in the supplemental material).

The two new groups of RCA phages, the CRP-6-type group and CRP-7-type group, are also ubiquitous in the ocean. The CRP-6-type group was more abundant than the CRP-7-type group in almost all cases (Fig. 4; see Fig. S3 in the supplemental material). In the upper layer of the coastal zone, the relative abundance of the CRP-6-type group was comparable with the HTVC019P-type group and the cyanophage T4-like group (Fig. 4). Among these four RCA phage groups, the CRP-7-type group was the least often present in the marine viromes but was still abundant and widely distributed in the ocean. The relative abundance of the CRP-7-type group was higher than those of roseophage N4-like and roseosiphoviruses in the vast majority of marine viromes (Fig. 4; see Fig. S3 in the supplemental material).

Interestingly, except for the HMO-2011-type group, other phage groups presented by RCA phages exhibited significantly higher abundance in the coastal waters than in the open ocean and intermediate regions (Fig. 4; see Fig. S3 in the supplemental material). In addition, all phage groups examined in this study were predominant in upper-ocean viromes (<200 m) (Fig. 4; see Fig. S3 in the supplemental material). In agreement, roseobacters have been reported to predominate along the coasts of temperate and polar environments (17, 18, 20–23), suggesting the tight cooccurrence and corelevant dynamic of RCA and RCA phages.

Our analysis clearly reveals the significant contribution of RCA phages to the diversity and abundance of marine viruses. As inferred from the “kill-the-winner” hypothesis, members of the abundant RCA *Roseobacter* lineage are subjected to a high level of density-dependent phage predation pressure. Therefore, it is not surprising that RCA phages constitute an important component of marine viral communities.

### Phage-host strategies.

Oceans contain highly abundant viruses, many of which are still waiting to be discovered. Similarly to SAR11, RCA represents a type of bacteria with reduced genome and oligotrophic adaptation (26). It is intriguing that slow-growing heterotrophic bacteria such as SAR11 (9, 52), SAR116 (10), and RCA are susceptible to infection by diverse podoviruses. Furthermore, these podoviruses are always highly ranked among the known phage groups (Fig. 4). Although many podoviruses have also been isolated from fast-growing roseobacters, such as Ruegeria pomeroyi DSS-3, *Sulfitobacter* spp., and Dinoroseobacter shibae DFL12 (27, 28, 30, 31), most of the existing roseopodoviruses are N4-like phages, which were the least present in marine viromes (Fig. 4) despite infecting multiple fast-growing *Roseobacter* lineages. The high abundance and diversity of podoviruses that infect SAR11, SAR116, and RCA bacteria suggest a unique phage-host strategy between the podoviruses and K-selected bacteria (slow growing but abundant) (11, 53, 54). This type of “kill-the-winner” strategy could be important to maintain the equilibrium of K-selected or specialist bacteria in the oceans.

Many easy-to-grow roseobacters are generalists (utilizing a variety of organic compounds) and have relatively larger genomes than RCA species (26). These bacteria can quickly respond to environmental changes and are considered to be r-selected bacteria (fast-growing but sporadically abundant) (53, 54). The acquisition of new genetic features is crucial for niche adaptation of r-selected roseobacters. Prophages are common in r-selected bacteria, while few or no prophages were found in K-selected bacteria (14, 26, 53, 55). Many siphoviruses have been isolated from the fast-growing roseobacters (27), and it is possible that these siphoviruses play a key role in horizontal gene transfer in fast-growing roseobacters. On the other hand, K-selected bacteria display reduced genomes and oligotrophic adaptation, utilizing a narrow range of organic compounds (specialists). Prophages are rarely found in K-selected bacteria. Although some podoviruses infecting SAR11, SAR116, and RCA bacteria contain an integrase (9, 10, 52), the relatively low content of integration sequences of HTVC019P-type pelagiphages in the Global Ocean Sampling (GOS) metagenomic database suggested that these phages infrequently lysogenize their hosts (52).

### Conclusions.

Here, we first reported and described seven phages that infect an important group of marine bacteria, the RCA *Roseobacter* lineage. RCA represents a group of difficult-to-culture, slow-growing, but abundant bacterioplankton. The successful isolation and cultivation of the RCA strains in the laboratory facilitated the discovery of novel roseophage groups. The seven RCA phage genomes elucidated in this study contribute to the expansion of the diversity of cultured marine roseophages. It is surprising to identify such diverse groups of podoviruses that infect these closely related RCA strains. Marine viral communities contain numerous novel phage types without representative isolates. Given the ecological significance of their hosts and their ubiquity in the ocean, our study provides valuable experimental model systems for investigating phage ecology and phage-host interactions.

The three RCA strains used in this study grow slowly in diluted medium and do not reach high cell densities compared to fast-growing roseobacters. Because many groups of abundant bacteria and archaea have not been cultivated, their interactions with viruses are still unknown. We advocate that additional efforts are needed to isolate phages that infect abundant but not-yet-cultivated bacterioplankton to better understand the viral diversity in the ocean and interpret marine viromic data sets.

## MATERIALS AND METHODS

### Cultivation of RCA strains, bacterial DNA extraction, and PCR.

The *Roseobacter* strains FZCC0023, FZCC0040, FZCC0042, and FZCC0083 were isolated on 13 May 2017 from the coastal water of Pingtan Island in China (latitude N25°26′, longitude E119°47′) using the dilution-to-extinction method (16). All strains were grown in an autoclaved seawater-based medium with excess vitamins (56) and amended with 1 mM NH_4_Cl, 100 μM KH_2_PO_4_, 1 μM FeCl_3_, and mixed carbon source (57). Cultures were incubated at 23°C. Genomic DNA of the RCA strains was extracted using a DNeasy Blood & Tissue kit (Qiagen, Valencia, CA, USA) following the manufacturer’s protocol. The 16S rRNA genes were amplified by PCR using the primers 16S-27F and 16S-1492R (58). The primers 16S-907F and 23S-189R were used for PCR amplification of 16S-23S rDNA intergenic transcribed spacer (ITS) sequences (59, 60). The 16S rRNA gene and ITS sequences of three strains were obtained by Sanger sequencing and assembled using ChromasPro (Technelysium Pty. Ltd., Tewantin, QLD, Australia). Experimental details are provided in Text S1 in the supplemental material.

### Source waters and RCA phage isolation.

The water samples used to isolate RCA phages were collected from a variety of coastal stations (Table 1). The seawater samples were filtered through 0.1-μm-pore-size filters and stored at 4°C prior to use. The phages were isolated according to a procedure detailed in a previous report (9). Briefly, 0.1-μm-filtered water samples were inoculated with exponentially growing host cultures and monitored for cell lysis using a Guava EasyCyte flow counter (Merck Millipore, Billerica, MA, USA). For cell lysis cultures, the presence of phage particles was further confirmed by epifluorescence microscopy with SYBR green I (Invitrogen, Eugene, OR, USA) (61). Purified RCA phage clones were obtained using the dilution-to-extinction method. The purity of the RCA phages was assessed by genome sequencing.

### Transmission electron microscopy.

RCA phage lysates were filtered through 0.1-μm-pore-size filters and then concentrated using Amicon Ultra centrifugal filters (30 kDa; Merck Millipore) followed by ultracentrifugation (Beckman Coulter, USA) at 50,000 × *g* for 2 h. Concentrated phages were absorbed onto copper grids in the dark and stained with 2% uranyl acetate for 2 min. Samples were observed using a Hitachi transmission electron microscope at 80 kV.

### Cross-infection experiments.

The cross-infectivity of seven RCA phages was tested in liquid medium against three RCA strains in duplicate. Exponentially growing cultures of three RCA strains were incubated with each RCA phage at a phage-to-host ratio of approximately 20. Cell lysis was monitored by using a Guava EasyCyte flow cytometer, and phage particles were enumerated by epifluorescence microscopy.

### Phage DNA preparation and genome sequencing.

Phage lysates (250 ml) were filtered through an 0.1-μm-pore-size Supor membrane and further concentrated using 30-kDa-molecular-weight (MW)-cutoff Amicon Ultra centrifugal filters. Phage DNA was extracted using the formamide extraction method (62). The genomes of the RCA phages were sequenced using the Illumina paired-end HiSeq 2500 sequencing approach at Beijing Mega Genomics Technology (Beijing, China). Quality filtration, removal of adapter and low-quality sequences, and *de novo* assembly of reads were performed using CLC Genomic Workbench 11.0.1 (Qiagen, Hilden, Germany) with the default settings. The gaps in phage genomes were closed by PCR amplification of the regions between the contigs, followed by conventional Sanger sequencing.

### Genome annotation and comparative genomic analysis.

Putative open reading frames (ORFs) were predicted from assembled RCA phage genomes by GENMARK (63), the RAST server (64), and manual inspection. tRNAs were detected using tRNAscan-SE (65). Putative biological functions were assigned to translated ORFs using BLASTp (amino acid identity ≥30%, alignment coverage ≥50%, and E value ≤1E−3) against the NCBI nonredundant (nr) database and the NCBI-RefSeq database in comparison with known proteins. PFAM and HHpred were also used to identify the protein families and distant protein homologs, respectively.

### Determination of CRP-3 integration sites.

The integration sites of CRP-3 were identified by following a strategy described in a previous study (52). Briefly, DNA of phage-infected cells was sequenced using the Illumina HiSeq 2500. The raw reads were quality filtered, trimmed, and mapped to the CRP-3 genome using CLC Genomic Workbench 11.0.1. Sequences that mapped to the CRP-3 genome were manually inspected to detect the phage-host hybrid sequences. The resulting hybrid sequences were analyzed to identify the integration sites and their locations on the host genomes. PCR primer sets (attL-F, TTCGGGACTGGAAGCATAC, and attL-R, CCTAAAGGCAGGAGGATACAC; attR-F, CAGAGCCTCTTTGTGATGGTC, and attR-R, AGGCACACTGGACTATACACAG) were designed based on the predicted *attL* and *attR* sites.

### Phylogenetic analysis.

Phylogenetic trees based on 16S rRNA gene and internal transcribed spacer (ITS) sequences were constructed using FastTree 2.1 (66) with the following settings: FastTree -gtr -nt -boot 1,000 sequences < alignment_file > tree_file. Sequences were aligned using MAFFT (67). Amino acid sequences of DNA polymerase were aligned and used for phylogenetic analysis. Alignments of DNA polymerase gene sequences were constructed with MUSCLE (68) and edited with Gblocks (69). Alignments were evaluated for the optimal amino acid substitution models using ProtTest (70), and the maximum likelihood tree was constructed by RAxML version 8.2.12 using the PROTGAMMA+WAGF model with a bootstrap of 500 replicates (71).

### Network analysis.

A total of 2,591 genomes (268,859 proteins) of bacterial viruses were downloaded from NCBI-RefSeq (v96), and an additional 788 viral contigs (≥20 kb, ≥20% shared genes with any RCA phage) were retrieved from metagenomic fosmids (5) and Global Ocean Viromes data sets (8, 72). All proteins were compared using all-versus-all BLASTp (E value ≤1E−5, bitscore ≥50), after which protein clusters were defined using the Markov clustering algorithm (MCL) (73). vConTACT2 (https://bitbucket.org/MAVERICLab/vcontact2) was used to calculate the similarities core between phage genomes and define the virus clusters (74).The network was visualized using Cytoscape 3.5.1, using an edge-weighted spring-embedded model. In the network, nodes represent phage sequences and the weight of each edge represents the distance between two phages based on the significance score.

### Phylogenetic placement of viromic reads assigned to HMO-2011-type DNA polymerase genes.

Amino acid sequences of HMO-2011-type DNA polymerase homologs were extracted from viral single-amplified genomes (vSAGs), Global Ocean Sampling (GOS) metagenomic sequences, and assembled Global Ocean Viromes (GOV) viral populations using BLASTp (E value ≤1E−3, coverage ≥80%). One hundred twenty-nine nearly full-length environmental viral DNA polymerase sequences were aligned with HMO-2011-type DNA polymerase sequences using MAFFT (67). A phylogenetic tree of this alignment was constructed by RAxML version 8.2.12 (71) with the following settings: raxmlHPC-PTHREADS -T 12 -f a -x 12345 -m PROTGAMMAWAGF -s alignment_file -# 100 -p 12345. The Pacific Ocean Virome (POV) reads assigned to the DNA polymerase genes of the HMO-2011-type phages were translated and placed on the phylogenetic tree using the pplacer v1.1 (75). The resulting tree phylogeny was visualized and manipulated using iTOL v4 (76).

### RCA phage fragment recruitment from marine virome.

Virome data sets from the Pacific Ocean Virome (POV), Moore Virome Project (MVP), Scripps Pier Virome (SPV), Indian Ocean Virome (IOV), and Malaspina Expedition virome (ME) were used for phage reciprocal metagenomic fragment recruitment analysis (see Data Set S1 in the supplemental material). The analysis was performed according to procedures detailed in a previous report (9); the detailed steps are as follows:

1. Each of the virome reads was searched as a query against the RefSeq viral database (release 88), 11 newly sequenced HTVC019P-type pelagiphages (52), and seven RCA phage genomes in this study using DIAMOND BLASTx with an E value cutoff of 1E−3 and a bitscore cutoff of 40.
2. The resulting hits were extracted from the virome data sets and used as queries for BLASTx, against a protein database containing the following:
  a. A subset of the viral genomes from RefSeq, excluding 2b.
  b. The viral genomes used in this study (see Data Set S1 in the supplemental material), including the 7 newly sequenced RCA phage genomes.
  c. A subset of the bacterial genomes from RefSeq (release 81).
3. Metagenomic sequences that returned a best-hit of the query genome from step 2b were identified and extracted from the metagenomic data sets.
4. The relative abundances of each phage group were calculated and normalized as the number of reads recruited to the group divided by the total number of kilobase pairs in average genome size and divided by the total number of million reads in the virome data set (reads mapped per kb of genome sequence per million of virome reads [RPKM]).

Due to the large amount of sequencing data in the Global Ocean Viromes (GOV) data sets (2.6 billion reads), a different strategy was used to determine the relative abundances of different phage groups in GOV. GOV reads were recruited onto the phage genomes using BLASTx with an E value cutoff of 1E−10. If a read was recruited to more than one phage genome, the read was associated with the phage that provided the highest bitscore.

### Data availability.

The 16S rRNA and ITS sequences have been deposited in the GenBank database under the accession numbers MK335922 to MK335924. The genome sequences of RCA phages have been deposited in the GenBank database under the accession numbers MK613343 to MK613349.
