# Supplementary material for: Diverse, Abundant, and Novel Viruses Infecting the Marine Roseobacter RCA Lineage
Source: mSystems. 2019 Dec 17;4(6):e00494-19. doi: 10.1128/mSystems.00494-19 (PMC6918029; doi:10.1128/mSystems.00494-19)
Supplement: TEXT S1 [file mSystems.00494-19-s0001.docx]

**Supplemental methods and results**

**The isolation and growth of RCA strains**

A seawater sample was collected in a polycarbonate bottle from the surface water of Pingtan coast (lat.’ N25°26’, long. E119°47) in May 2017. The seawater sample was enumerated with a Guava EasyCyte flow cytometer (Merck Millipore, Billerica, MA, USA) and then diluted with autoclaved seawater-based media. Diluted sample were dispensed into 24-well microplates with a final inoculation density of 3 cells per well and incubated at 20 °C in the dark. After four weeks, each well was screened for cell growth by Guava EasyCyte flow cytometer, and wells with at least 10^5^ cells ml^-1^ were used for further 16S rRNA gene analyses. 400 μl of the cultures were centrifuged at 12, 000 rpm at 4 °C for 30 min. The cell pellet was then resuspended in 20 μl TE buffer. Template DNA were prepared for PCR by heating at 95°C for 10 min and then quickly chilled on ice. The16S rRNA genes were amplified by PCR using the primers 16S-27F and 16S-1492R. Positive PCR products were sequences and analyzed. Of the total 408 wells, 106 displayed cell growth-positive.16S rRNA genes analysis showed that three isolates belong to the *Roseobacter* RCA lineage. The growth curves of three RCA strains are shown in Fig.S1. FZCC0023, FZCC0040 and FZCC0040 reached maximum cell densities of 1.2×10^7^ cells ml^-1^, 2.5×10^7^ cells ml^-1^ and 0.65×10^7^ cells ml^-1^ (n=3), respectively. All three strains had a phase of rapid logarithmic growth (maximum growth rate of FZCC0023, FZCC0040 and FZCC0040 are 2.07 d^-1^, 2.74 d^-1^ and 2.27 d^-1^, respectively), after which their growth rates decreased significantly (Fig.S1c). All three RCA strains can neither grow in artificial seawater based medium nor form visible colonies on solidified media.

**Extinction dilution procedure used for phage purification**

Bacteria were cultivated in 24 well microplates at 20 °C in the dark. The initial inoculation density in each well was about 10^5^ cells ml^-1^. Phage lysates were diluted with culture medium and added into host cultures. Each well was estimated to contain 10, 5 or 1 phage particles. After three weeks, each well was screened for cell lysis by using a Guava EasyCyte flow cytometer. Phage lysate were transferred from the most diluted lysis culture and the dilution procedure was repeated.
