# Supplementary figures and images for: Diverse, Abundant, and Novel Viruses Infecting the Marine Roseobacter RCA Lineage
Source: mSystems. 2019 Dec 17;4(6):e00494-19. doi: 10.1128/mSystems.00494-19 (PMC6918029; doi:10.1128/mSystems.00494-19)

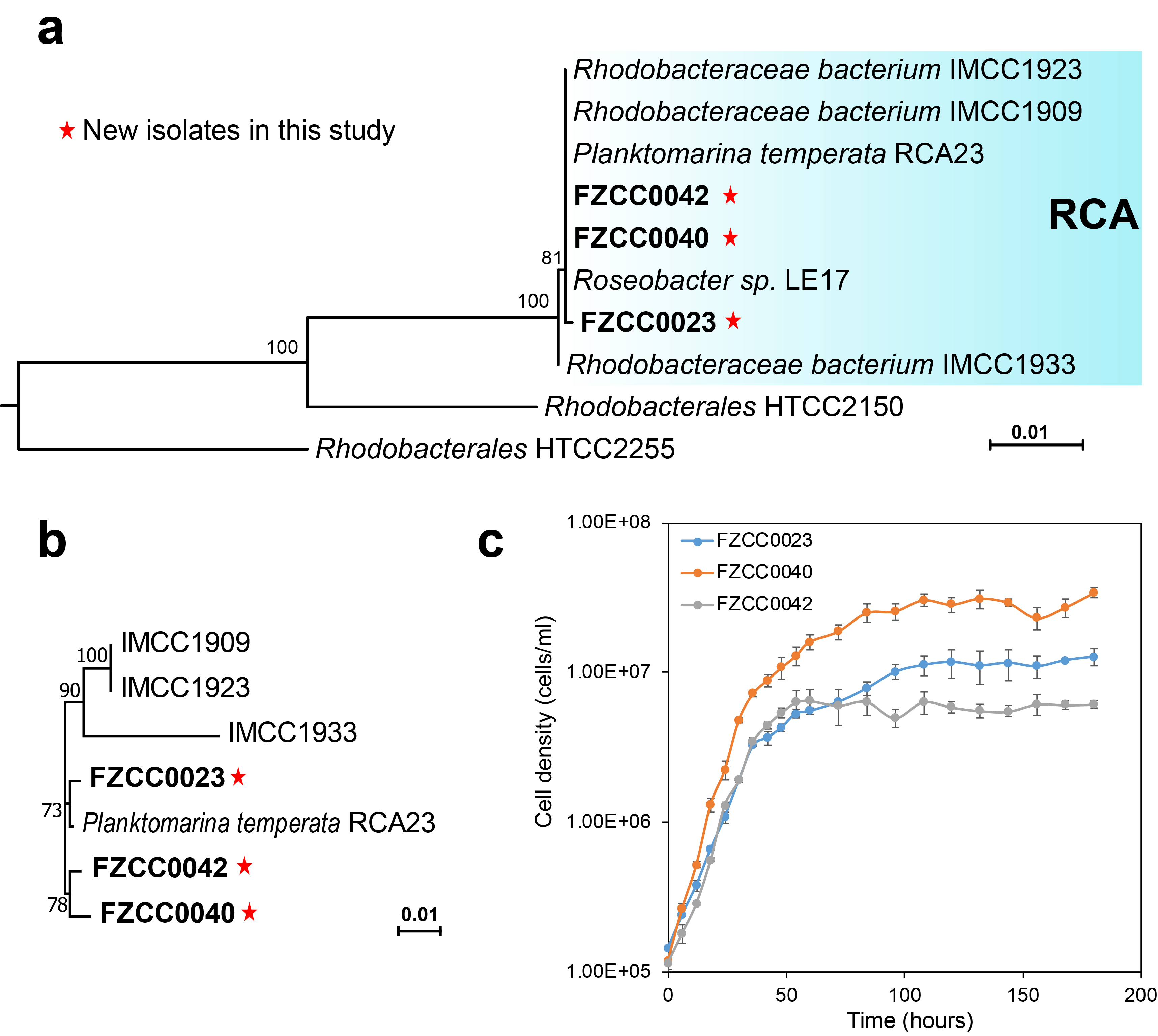

Supplement: FIG S1 [file mSystems.00494-19-sf001.tif]

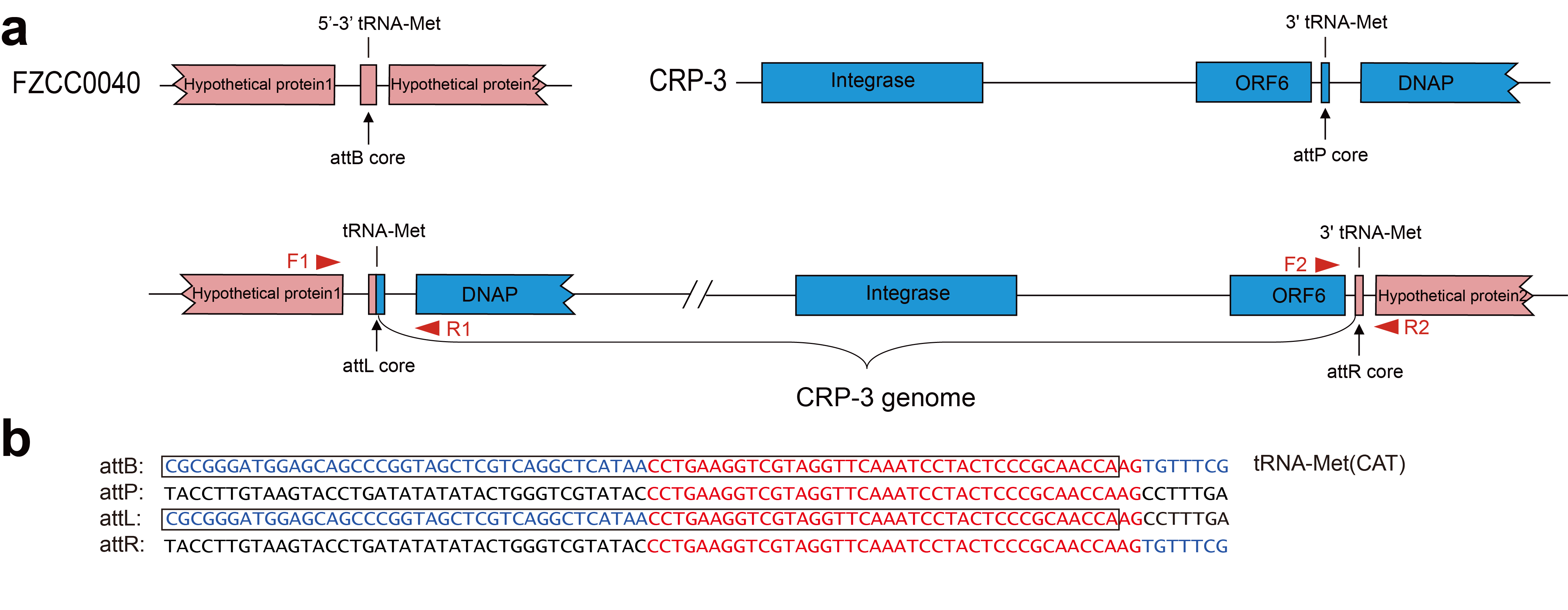

Supplement: FIG S2 [file mSystems.00494-19-sf002.tif]

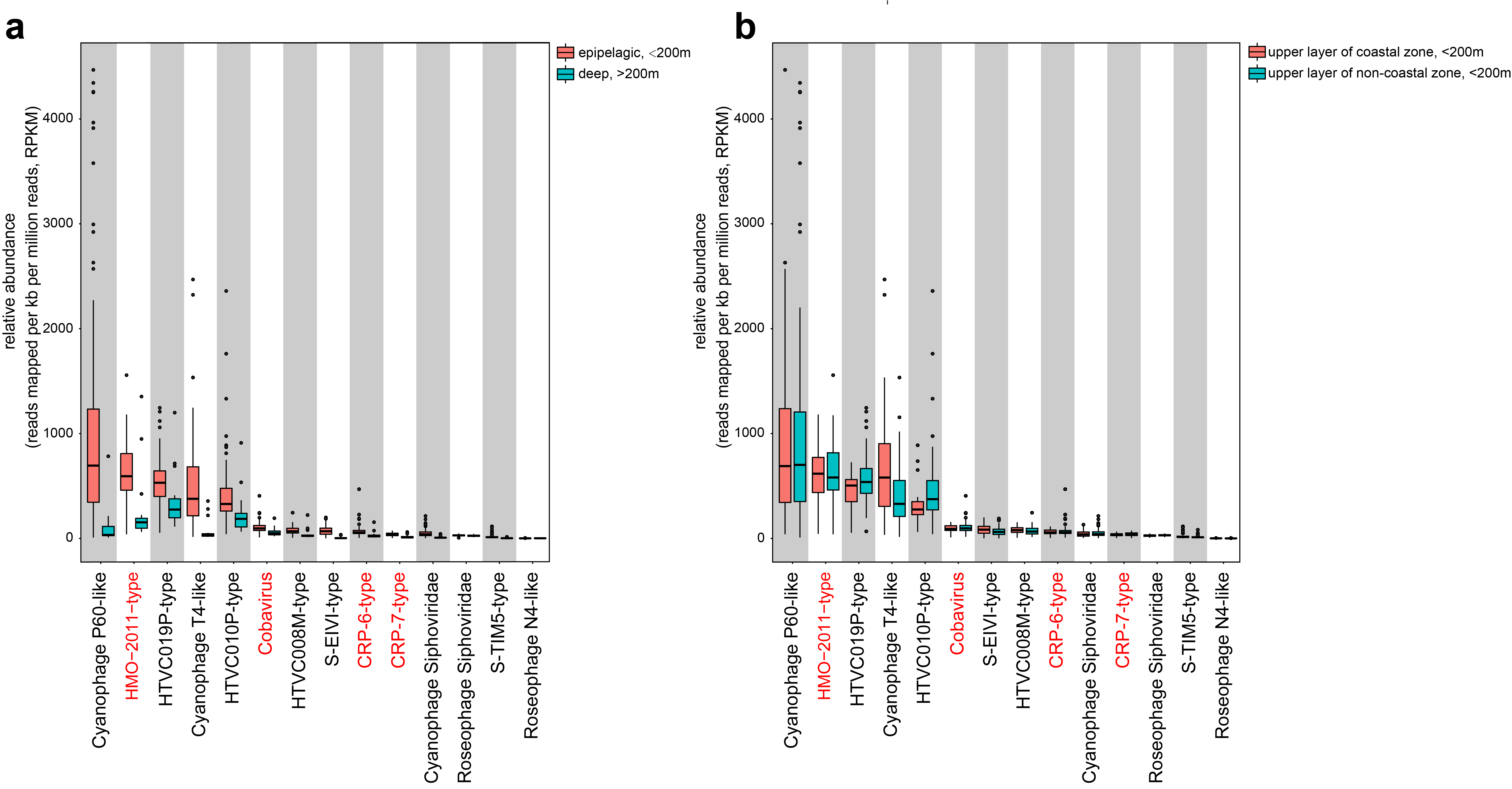

Supplement: FIG S3 [file mSystems.00494-19-sf003.tif]

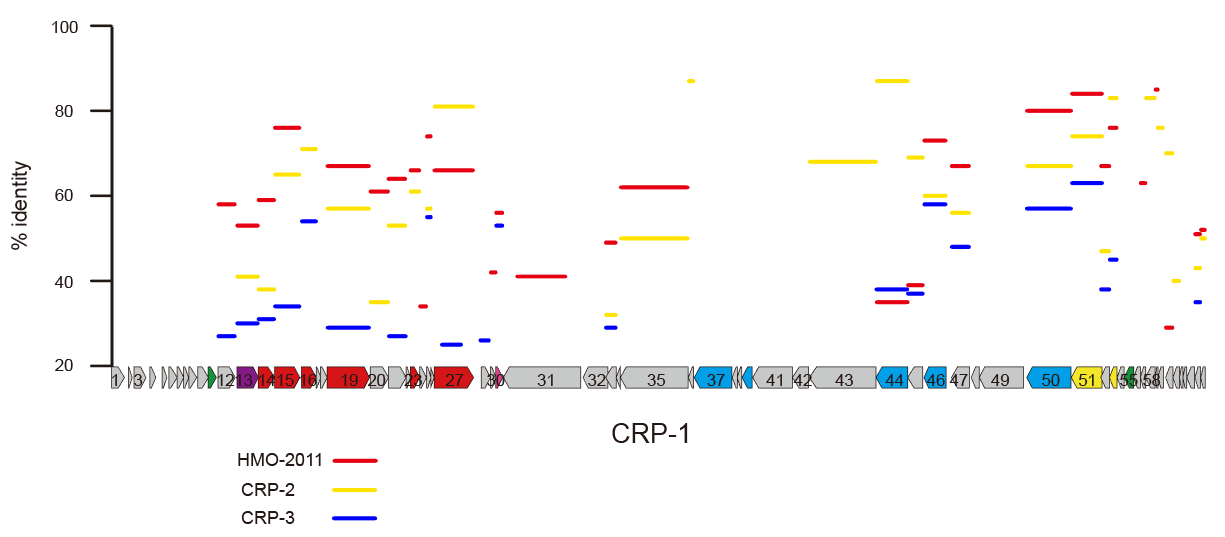

Supplement: FIG S4 [file mSystems.00494-19-sf004.tif]

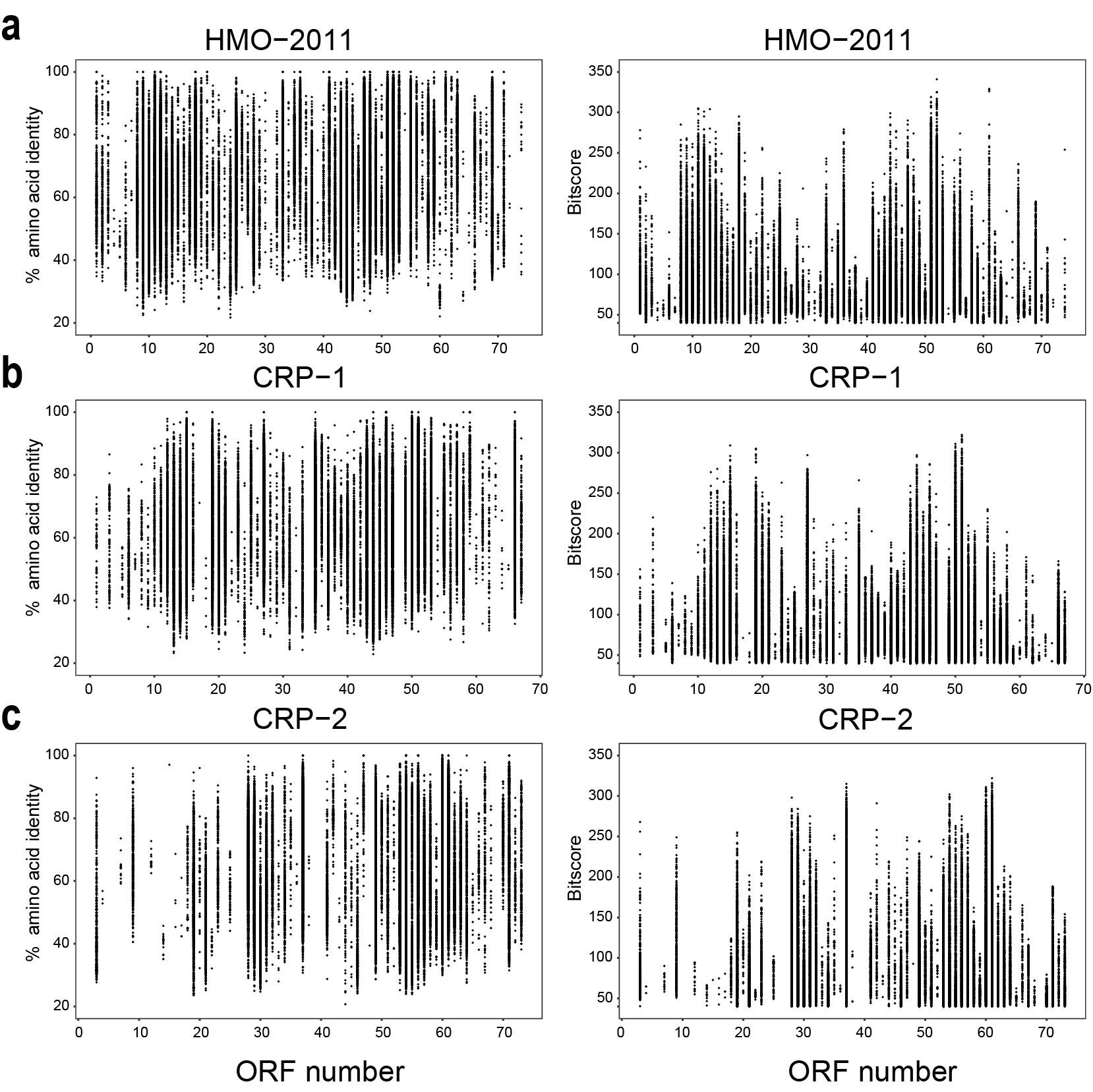

Supplement: FIG S5 [file mSystems.00494-19-sf005.tif]

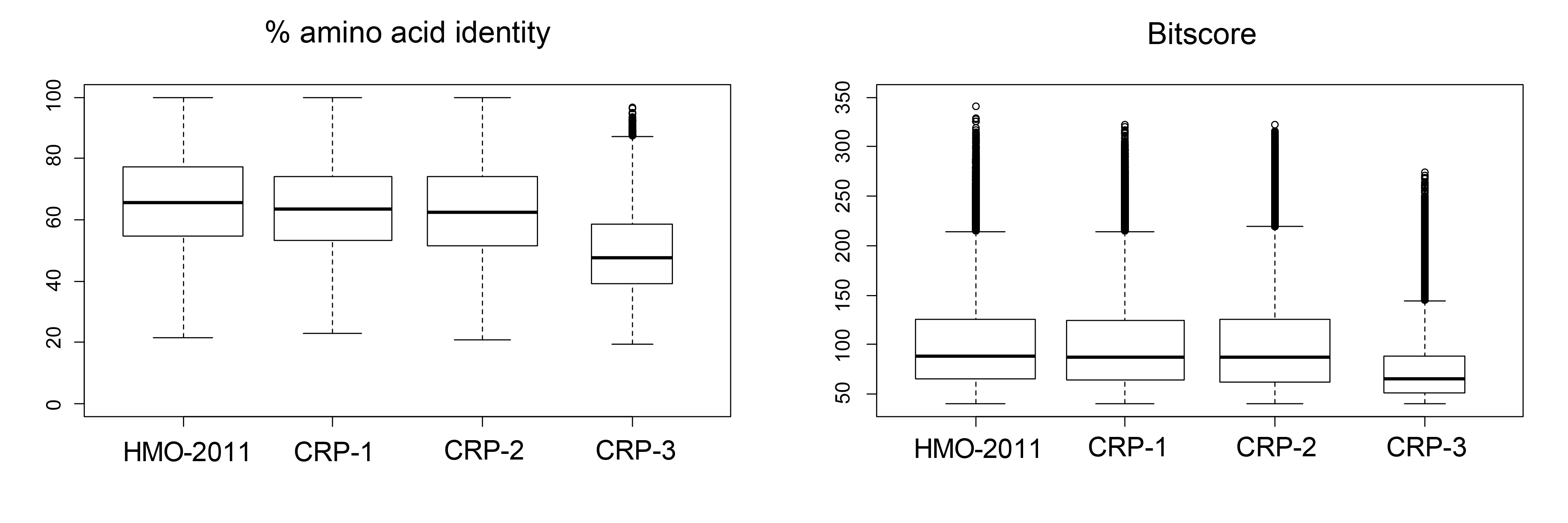

Supplement: FIG S6 [file mSystems.00494-19-sf006.tif]
