# Supplementary material for: Diverse, Abundant, and Novel Viruses Infecting the Marine Roseobacter RCA Lineage
Source: mSystems. 2019 Dec 17;4(6):e00494-19. doi: 10.1128/mSystems.00494-19 (PMC6918029; doi:10.1128/mSystems.00494-19)
Supplement: TABLE S1 [file mSystems.00494-19-st001.docx]

| Phage | MazG | Methyltransferase | PhoH | Glutaredoxin | GNAT | GCT | ThyX | RNR |
| --- | --- | --- | --- | --- | --- | --- | --- | --- |
| CRP-1 | ORF30 |  |  |  |  |  | ORF16 | ORF27 |
| CRP-2 |  | ORF9 | ORF42 | ORF38 |  |  | ORF23 | ORF37 |
| CRP-3 | ORF16 |  |  |  |  |  | ORF4 | ORF17 |
| CRP-4 | ORF22 |  | ORF27 | ORF25 | ORF41 |  | ORF10 | ORF24 |
| CRP-5 | ORF23 |  | ORF28 | ORF26 | ORF41 |  | ORF16 | ORF25 |
| CRP-6 | ORF24 |  | ORF30 | ORF29 |  | ORF2 | ORF8 | ORF28 |
| CRP-7 | ORF9 |  |  | ORF26 |  |  |  | ORF44,46 |

^a^ThyX: thymidylate synthase.

^b^PhoH: phosphate starvation-inducible protein.

^c^MazG: nucleotide pyrophosphohydrolase domain protein.

^d^RNR: adenosylcobalamin-dependent ribonucleoside-triphosphate reductase.

^e^GNAT: GCN5-Related N-Acetyltransferases Acetyltransferase family.

^f^GCT: glycerol-3-phosphate cytidylyltransferase (GCT).
